# Health Status of Nonemergency Patients in the Emergency Department Using the EQ-5D

**DOI:** 10.1155/2024/7880345

**Published:** 2024-03-31

**Authors:** Min Dai, Jingyuan Jiang, Lingjun Jiang, Jin Zhou, Lei Ye

**Affiliations:** ^1^Department of Emergency Medicine, West China Hospital, West China School of Nursing, Sichuan University, Chengdu, China; ^2^Institute of Disaster Medicine, Sichuan University, Chengdu, China; ^3^Nursing Key Laboratory of Sichuan Province, Chengdu, China; ^4^Nursing Department of West China Hospital, Sichuan University, Chengdu, China; ^5^Department of Emergency and Trauma Nursing, West China Nursing School, Sichuan University, Chengdu, China

## Abstract

**Background:**

Emergency department (ED) overcrowding is influenced by several factors including the hospital's capacity, staff, patient discharges, and community resources. The number of annual ED visits has increased, with patients' medical needs exceeding emergency capacity, resulting in a widespread concern about emergency room overcrowding. Nonemergency patients tend to use large amounts of emergency medical resources, which is one reason for ED overcrowding. Most patients consider their medical cases urgent, whereas medical professionals consider many cases to be nonemergency. Only a few studies have examined self-rated health among nonemergency patients.

**Methods:**

This cross-sectional study was conducted in the ED of a tertiary hospital in China using the European Quality of Life Five-Dimensional Questionnaire to investigate the health status of nonemergency patients.

**Results:**

Among the 545 respondents, 246 (45.14%) self-assessed their health as excellent, 186 (34.13%) as very good, 70 (12.84%) as good, 32 (5.87%) as average, and 11 (2.02%) as poor. Problems related to pain/discomfort were reported by 317 (58.17%) participants, 214 (39.27%) responded that they had problems related to daily activities, 212 (38.90%) responded that they felt anxious or depressed, 211 (38.35%) responded that they had problems related to self-care, and some or extreme problems related to mobility were stated by 193 people (35.41%).

**Conclusions:**

Nonemergency patients generally reported good health. Pain/discomfort was the most significant factor affecting the health of nonemergency patients, followed by limitation of daily activities. The duration of illness onset and self-rated health status were common factors influencing the health status of nonemergency patients. This trial is registered with ChiCTR1900023578.

## 1. Introduction

Emergency department (ED) overcrowding is multifactorial [1]. It is related to the hospital's capacity, staff, patient discharge, community resources, and other factors. The number of ED visits has been increasing year by year, and the patient's medical needs can exceed the emergency capacity, causing ED crowding to be a phenomenon of widespread concern globally [2–4]. A study has reported that 32.7% of ED visits were for nonemergency health issues [5]. Another study conducted at the ED of King Abdullah bin Abdulaziz University Hospital reported a nonemergency attendance rate of 61.4% [6], and a cross-sectional study in Iran showed that nonemergency visits accounted for 64.6% of ED visits [7]. Wang et al. [8] reported that the average percentage of true emergencies in EDs in China is very low. Long queues in the ED have become the norm in the EDs of many large general hospitals, and the utilization of emergency resources by nonemergency patients is also very common. Emergency medical treatment for nonemergency patients is also an important reason for ED crowding and has greatly increased the operating pressure on the ED system [9]. Researchers have attempted to explore nonemergency patients and their reasons for ED presentation [5, 10]. There are various reasons for nonemergency patients using the ED, which can be summarized into three aspects: the patient's own perceived need for emergency medical treatment, the inability of alternative medical service resources to meet their diagnosis and treatment needs, and the convenience of emergency resources [11–17]. These phenomena indicate that, on the one hand, nonemergency patients adopt emergency treatment due to patients, family members, or friends overestimating the severity of the disease and expect reassurance from the ED due to concerns about disease progression. Primary care facilities, on the other hand, lack radiology or laboratory testing and have difficulty accepting outpatient appointments.

The EuroQoL five-dimensional questionnaire (EQ-5D) is a short, reliable, and validated instrument commonly used to assess health status [18]. The EQ-5D is easy to use and places few demands on the cognitive function of study subjects. Yang and Tang [19] used the EQ-5D to measure health-related quality of life and its influencing factors in patients with chronic diseases, and Ping et al. [20] used the EQ-5D to assess health-related quality of life during the COVID-19 pandemic in China. The health status of nonemergency patients reflects their overall state of physical, mental, social, spiritual, and personal functioning. Therefore, this study used the EQ-5D to investigate the health status of nonemergency patients in the ED, with the aim of providing a reference for clinical practice in the scientific management of this group.

## 2. Methods

### 2.1. Study Design and Setting

This cross-sectional study (registration number: ChiCTR1900023578) was conducted in the ED of the West China Hospital of Sichuan University, a 4300-bed tertiary teaching hospital with 250,000 ED visits per year. This study was scheduled to be conducted between June 1 and 20, 2019. Participants who were defined as nonemergency visits were recruited by trained triage nurses 24 h a day using convenience sampling. After the triage procedure, the target patients were briefed on the study protocol. A questionnaire survey was administered to the patients who agreed to participate. Participants were instructed to use their mobile phone to scan QR codes for the survey. Prior to the study, several training sessions were conducted to ensure proper implementation of the survey. A 60 min group training session was provided to all triage nurses to promote a consistent understanding of the study objectives and survey instruments.

### 2.2. Participants

Generally speaking, nonemergency patients refer to those who have no acute symptoms, no or few complaints of discomfort, require fewer emergency resources, and whose treatment can be delayed for several hours. For patients attending the ED, their emergency status was determined based on triage standards. Patients with the lowest emergency levels, IV and/or V, were defined as nonemergency patients [21, 22]. An experienced advanced triage nurse identified nonemergency patients, including those of different age groups, sexes, and ethnicities. Patients who refused to participate, did not speak Chinese, or were mentally unable to participate were excluded. Patients for whom there were missing data were also excluded. For patients <14 years of age, their caregivers were asked to complete the EQ-5D. The final number of participants was 545. To ensure anonymity, all patient data were accessible only to the research team. The sample size was calculated based on the proportion of patients who had tried alternative services before their ED visits. Assuming that 60% of nonemergency patients try alternative services [23], a sample of 425 patients is required. Assuming a sampling error of 0.1 and a nonresponse rate of 20%, the target was a total of 510 patients. Some data were missing due to the incomplete completion of the questionnaire. This lack of data could be systematic (nonrandom) in that certain patient groups may have been unable to fully complete the questionnaire.

### 2.3. Instrument

Developed in 1990 by the EuroQoL Group, a voluntary multinational collaboration of European researchers, the EQ-5D is applicable to a wide range of health conditions and includes single indicator values of health status and self-rating visual analog scales (VAS) available in population health surveys. To check the validity of the initial questionnaire, five local experts with more than 10 years of ED work experience and at least associate senior professional titles completed the questionnaire. The content validity of the questionnaire was good, with an overall content validity index score of 0.8854. After appropriate modifications, the questionnaire was pretested on 35 patients to ensure readability. The final questionnaire collected information on sociodemographic characteristics, medical resource allocation, health status, and the EQ-5D (Table 1). Socioeconomic and demographic information included sex, age, ethnicity, employment status, education level, marital status, income, health insurance, and living conditions. Medical resource allocation included the distance to the emergency room, nearest medical service institution, distance to the nearest medical service institution, mode of transportation for emergency medical treatment, and whether the institution was considered to meet health needs in the near future. Health status included the onset time, presence of chronic diseases, self-evaluation of health status, and self-evaluation of urgency. The EQ-5D [18, 24, 25] consisted of two parts: the health description system and the EQ visual analogue scales (EQ-VAS). The health description system included five dimensions: mobility, self-care, daily activities, pain/discomfort, and anxiety/depression. Each dimension was divided into three levels: no difficulty, difficulty, and extreme difficulty. Participants were asked to answer five questions about whether they had a problem and the severity of the problem. The EQ-VAS is a 20-cm-long vertical visual scale. The score ranges from 0 to 100, with the top score of 100 representing the best health status in mind, “no pain” or “complete comfort,” and the bottom score of 0 representing the worst health status in mind, “worst pain” or “complete discomfort.” Respondents were asked to mark the point on a straight line where they felt pain or discomfort. We divided the VAS score into five ranges: 81–100, 61–80, 41–60, 21–40, and 0–20 points, with higher scores indicating better overall health. Informed consent was obtained from all participants after the study objectives were explained and anonymity and confidentiality were ensured.

### 2.4. Statistical Analysis

All statistical analyses were performed using SPSS version 20.0. Descriptive statistics were used to describe the patient characteristics. Chi-squared tests and multivariate logistic regression analyses were performed. Statistical significance was set at *P* < 0.05. Variables with significant differences in the chi-square test (two-sided *P* < 0.05) were included in the multivariate regression analysis.

### 2.5. Ethics Statement

The study protocol was reviewed and approved by the Institutional Review Board of Sichuan University Hospital, China. All the questionnaires were collected anonymously.

## 3. Results

### 3.1. Participant Characteristics

During the study period, 10,450 emergency room visits occurred, 980 of which were defined as nonemergencies. Of these patients, 187 declined to participate, and 190 patients were unsuccessfully enrolled (186 patients were missed when the ED was at its busiest, and 4 patients were excluded due to aggressive language and behavior). Of the 603 patients who participated in the study, 58 were excluded because they did not complete all survey questions. Table 1 presents the characteristics of the study participants. Among the 545 respondents, 274 were female (50.28%), 217 were aged 19–44 years (39.81%), 514 were Han ethnicity (94.31%), 219 were employed (40.18%), and 338 were educated to high school level or lower (62.02%).

### 3.2. EQ-VAS Scores

Table 2 shows the health status scores of nonemergency patients. Regarding the question, “How is your overall health today?,” the self-rated scores on the EQ-VAS were 81–100 points by 246 people (45.14%), 61–80 points by 186 people (34.13%), 41–60 points by 70 people (12.84%), 21–40 points by 32 people (5.87%), and 0–20 points by 11 people (2.02%).

### 3.3. EQ-5D Responses

Of the 545 participants, 193 (35.41%) stated that they had some or extreme problems related to mobility, 211 (38.35%) responded that they had problems related to self-care, 214 (39.27%) responded that they had some or extreme problems related to daily activities, 317 (58.17%) said they had some or extreme problems related to pain/discomfort, and 212 (38.90%) responded that they felt anxious or depressed. Pain/discomfort was the most serious factor affecting the health status, followed by limitation of daily activities (Table 3).

### 3.4. Factors Affecting EQ-5D Response

There were significant associations between age, occupation, disease duration, self-rated health status, and mobility (Table 4). Older age (odds ratio (OR) = 1.341, *P*=0.009), “other” employment status (retired, unoccupied, and student) (OR = 1.662, *P*=0.013), longer illness duration (OR = 1.153, *P* < 0.001), and lower self-rated health status (OR = 0.694, *P*=0.009) were associated with lower mobility.

There were significant associations between ethnicity, educational level, mode of transportation, disease duration, self-rated health status, and self-care ability. Ethnic minorities (OR = 2.448, *P*=0.029), high school or lower education (OR = 0.479, *P* < 0.001), transportation by ambulance (OR = 18.709, *P*=0.007), longer illness duration (OR = 1.508, *P* < 0.001), and lower self-rated health status (OR = 0.662, *P* < 0.001) were associated with poorer self-care.

There were significant associations between occupation, mode of transportation, illness duration, self-rated health status, self-rated urgency, and daily activities. “Other” employment status (retired, unoccupied, and student) (OR = 1.609, *P*=0.016), transportation by ambulance (OR = 7.896, *P*=0.013), longer illness duration (OR = 1.420, *P*=0.002), lower self-rated health status (OR = 0.592, *P* < 0.001), and higher self-evaluated urgency (OR = 1.420, *P*=0.010) were associated with poorer daily life activities.

Transportation mode, illness duration, and self-rated health status were significantly associated with pain and discomfort. Transportation by bus/subway (OR = 0.375, *P*=0.013) and private car (OR = 0.421, *P*=0.026), longer illness duration (OR = 1.553, *P* < 0.001), and lower self-rated health status (OR = 0.755, *P*=0.028) were associated with worse reported pain/discomfort. Private car users experienced higher pain/discomfort than bus/subway users.

There were significant associations between illness duration, self-rated health status, and anxiety/depression. Longer illness duration (OR = 1.716, *P* < 0.001) and lower self-rated health status (OR = 0.611, *P* < 0.001) were associated with more anxiety and depression.

## 4. Discussion

### 4.1. Nonemergency Patients Report That Their Overall Health Status Is Good

Consistent with the results of many studies in this field, e.g., [26], our results showed that most nonemergency patients were <45 years of age (369, 67.71%), with good health status (432, 79.2%) and low chronic disease morbidity (123, 22.57%). Our study found that nonemergency patients in the ED generally reported good health. This result is consistent with our expectations because nonemergency patients often seek care for nonserious acute conditions. Additionally, nonemergency patients tend to subjectively evaluate their health status as good because of their relatively low experience of acute illness and better quality of daily life. However, although nonemergency patients reported good health, this does not imply that they do not require attention or treatment. Instead, we should focus on chronic disease management and health maintenance to prevent disease progression and improve quality of life.

### 4.2. Pain/Discomfort Is the Most Important Factor Affecting the Health Status of Nonemergency Patients, Followed by Daily Activities

Our research found that among the five dimensions of EQ-5D in nonemergency patients, the factor that most significantly affected the health status was pain or discomfort. Yang et al. [27] reported that the discomfort dimension had the greatest impact on health status, which is consistent with the present results. This shows that even if patients do not have an acute illness, pain or discomfort remains an important factor affecting their health and quality of life. Pain may limit a patient's ability to perform daily activities and affect work, social, and family life. Our study found that the second most common dimension, limited daily activities, was also an important factor affecting the health status of nonemergency patients. This may be because of pain, limited physical function, or other chronic health issues. Limitations in daily activities can affect patients' quality of life and increase their risk of anxiety and depression. However, another study reported that the second most common dimension was anxiety/depression [20], which is inconsistent with the current results. One possible explanation is that nonemergency patients may have chronic illnesses or underlying risk factors that limit their daily activities.

### 4.3. Analysis of Factors Affecting the Health Status of Nonemergency Patients

The study found that illness duration and self-rated health status were common influencing factors for the five dimensions of health status of nonemergency patients in the ED. Prolonged disease duration showed a trend consistent with the deterioration of multiple health dimensions. Long-term illness may lead to a reduced quality of life, lower self-rated health, and exacerbation of various health problems. In particular, pain/discomfort and anxiety/depression may worsen over time, suggesting that long-term illness has a negative impact on patients' mental health [28]. There is a complex relationship between patients' evaluations of their health status and health behaviors. Patients with lower self-rated health may neglect self-care and report lower levels of daily activity. The interaction between self-rated health status and health behaviors requires greater attention in treatment and rehabilitation programs. Overall, these findings highlight the important influence of illness duration and self-rated health on the health status of nonemergency patients presenting to the ED.

The study found that the higher the educational level, the stronger the self-care ability. This may be because higher education levels ensure a better ability to acquire health knowledge and increase health awareness. Well-educated people are generally likely to have higher incomes and more social resources, and they can access better medical care including quality, accessibility, and efficiency. The study found that patients' medical choices may be influenced by their experiences and that their experience with medical services increases their expectations when seeking urgent care. The Han people's ability to take care of themselves was higher than that of ethnic minorities. China is a multiethnic country dominated by the Han people. Due to a lack of social resources such as health education and medical services, ethnic minorities are often regarded as vulnerable groups. Minority participants' frequent emergency care use for nonemergency conditions may be due to their lower levels of health literacy or difficulty in accessing general health services [29]. Ethnicity has been shown to be a relevant factor affecting health service utilization patterns; therefore, we consider that ethnicity is an important demographic factor because cultural differences among different ethnic groups may affect health service utilization.

### 4.4. Limitations

This study had several limitations. First, the sample size was small because the patients were recruited from only one hospital. Therefore, further studies are needed to confirm the generalizability of the findings. Secondly, misclassification of the emergency level may have occurred, such as grade 4 patients being incorrectly registered as grade 3 patients. Additionally, high nonparticipation rates among eligible participants may have affected the representativeness of the sample. For children under 14 years of age, caregivers completed questionnaires on their behalf, and the agent may not have fully understood or reported the child's feelings, behaviors, or needs. The agent's perspective may be influenced by their own beliefs, expectations, and experiences, which may not fully align with the child's actual experience. Other potential limitations associated with the mode and timing of administration should be considered in future studies.

## 5. Conclusions

This study provides a multidimensional understanding of the health status of nonemergency patients attending the ED, including mobility, self-care, daily activities, pain/discomfort, and anxiety/depression. People triaged as nonemergency reported generally good health status, but this was poorer among people who had a longer duration of illness. Health status deficits most commonly affected the pain and discomfort domain. Future research and clinical practice should explore the complex relationships among these factors and develop more effective interventions to promote the overall health and well-being of patients. When evaluating and treating such patients, these aspects must be comprehensively considered to provide effective management and to improve their quality of life and health.

## Figures and Tables

**Table 1:** Basic information of respondents.

Variable		Number	Composition ratio (%)
Sex	Male	271	49.72
	Female	274	50.28

Age (years)	≤18	152	27.89
	19–44	217	39.82
	45–65	123	22.57
	>65	53	9.72

Ethnicity	Han	514	94.31
	Ethnic minority	31	5.69

Employment status	Employed	219	40.18
	Other (retired, unemployed, student)	326	59.82

Education	High school or lower	338	62.02
	College or higher	207	37.98

Marital	Married	271	49.72
	Other	274	50.28

Income (RMB per month)	≤1800	111	20.37
	1801–5000	211	38.72
	500–8000	88	16.15
	>8000	135	24.77

Medical insurance	Uninsured	46	8.44
	One kind of insurance	421	77.25
	Two or more kinds of insurance	78	14.31

Living situation	Living alone	65	11.93
	Live with others	480	88.07

Distance to ED	15 min	60	11.01
	15–30 min	156	28.62
	>30 min	329	60.37

Nearest health service	Clinic/pharmacy	153	28.07
	Community hospital	198	36.33
	District/municipal general hospital	153	28.07
	Provincial hospital	41	7.52

Distance to nearest health service	15 min	266	48.81
	15–30 min	166	30.46
	>30 min	113	20.73

Travel mode	Walking	43	7.89
	Bus/subway	163	29.91
	Taxi	143	26.24
	Private car	177	32.48
	Ambulance	19	3.49

Seek medical advice nearby	No	56	10.28
	Not sure	263	48.26
	Yes	226	41.47

Do nearby institutions meet needs?	No	100	18.35
	Partly	367	67.34
	Yes	78	14.31

Illness duration	<1 day	244	44.77
	1-2 days	67	12.29
	>3 days	234	42.94

Presence of chronic disease	No chronic disease	422	77.43
	One or more chronic diseases	123	22.57

Self-assessment of urgency	Nonemergency (0–60)	186	34.13
	Emergency (61–80)	241	44.22
	Critical (81–100)	118	21.65

Total		545	100.00

**Table 2:** Health status scores of nonemergency patients.

Variable	Number	Composition ratio (%)
Self-rated health		
0–20 points	11	2.02
21–40 points	32	5.87
41–60 points	70	12.84
61–80 points	186	34.13
81–100 points	246	45.14

**Table 3:** EQ-5D distribution characteristics.

EQ-5D items	No problems	Some problems	Extreme problems
Mobility	352 (64.59%)	126 (23.12%)	67 (12.29%)
Self-care	334 (61.28%)	156 (28.62%)	55 (10.09%)
Usual activities	331 (60.73%)	160 (29.36%)	54 (9.91%)
Pain/discomfort	228 (41.83%)	243 (44.59%)	74 (13.58%)
Anxiety/depression	333 (61.10%)	171 (31.38%)	41 (7.52%)

**Table 4:** Factors affecting EQ-5D response.

Dependent variable	Independent variable	*P* value	Odds ratio	95% confidence interval
Mobility	Age	0.009	1.341	1.076–1.672
	Employment status	0.013	1.662	1.115–2.479
	Onset time	<0.001	1.513	1.210–1.892
	Self-rated health	0.009	0.694	0.528–0.912

Self-care	Ethnicity	0.029	2.448	1.095–5.472
	Education	<0.001	0.479	0.318–0.720
	Travel mode	0.006		
	Ambulance	0.007	18.709	2.204–158.843
	Onset time	<0.001	1.508	1.212–1.875
	Self-rated health status	<0.001	0.622	0.481–0.806

Usual activities	Employment status	0.016	1.609	1.091–2.374
	Travel mode	0.004		
	Ambulance	0.013	7.896	1.55–40.001
	Onset time	0.002	1.420	1.142–1.765
	Self-rated health status	<0.001	0.592	0.455–0.770
	Self-assessment of urgency	0.010	1.420	1.088–1.853

Pain/discomfort	Travel mode	0.019		
	Bus/subway	0.013	0.375	0.173–0.813
	Private car	0.026	0.421	0.196–0.903
	Onset time	<0.001	1.553	1.264–1.910
	Self-rated health status	0.028	0.755	0.588–0.971

Anxiety/depression	Onset time	<0.001	1.716	1.398–2.107
	Self-rated health status	<0.001	0.611	0.478–0.781

## Data Availability

The data that support the findings of this study are available from the corresponding author upon reasonable request.

## Additional Points

*Clinical Trials Registry*. This study was preregistered at the Chinese Clinical Trial Registry; the registration number is ChiCTR1900023578.
